# Older Adults’ Experiences Using a Commercially Available Monitor to Self-Track Their Physical Activity

**DOI:** 10.2196/mhealth.5120

**Published:** 2016-04-13

**Authors:** Siobhan K McMahon, Beth Lewis, Michael Oakes, Weihua Guan, Jean F Wyman, Alexander J Rothman

**Affiliations:** ^1^ University of Minnesota School of Nursing Minneapolis, MN United States; ^2^ University of Minnesota, School of Kinesiology Minneapolis, MN United States; ^3^ University of Minnesota, School of Public Health Minneapolis, MN United States; ^4^ University of Minnesota, Department of Psychology Minneapolis, MN United States

**Keywords:** Aged, Mobile Health, Self-Appraisal, Physical Activity, Motivation, Monitoring, Ambulatory, Wearables

## Abstract

**Background:**

Physical activity contributes to older adults’ autonomy, mobility, and quality of life as they age, yet fewer than 1 in 5 engage in activities as recommended. Many older adults track their exercise using pencil and paper, or their memory. Commercially available physical activity monitors (PAM) have the potential to facilitate these tracking practices and, in turn, physical activity. An assessment of older adults’ long-term experiences with PAM is needed to understand this potential.

**Objective:**

To assess short and long-term experiences of adults >70 years old using a PAM (Fitbit One) in terms of acceptance, ease-of-use, and usefulness: domains in the technology acceptance model.

**Methods:**

This prospective study included 95 community-dwelling older adults, all of whom received a PAM as part of randomized controlled trial piloting a fall-reducing physical activity promotion intervention. Ten-item surveys were administered 10 weeks and 8 months after the study started. Survey ratings are described and analyzed over time, and compared by sex, education, and age.

**Results:**

Participants were mostly women (71/95, 75%), 70 to 96 years old, and had some college education (68/95, 72%). Most participants (86/95, 91%) agreed or strongly agreed that the PAM was easy to use, useful, and acceptable both 10 weeks and 8 months after enrolling in the study. Ratings dropped between these time points in all survey domains: ease-of-use (median difference 0.66 points, *P*=.001); usefulness (median difference 0.16 points, *P*=.193); and acceptance (median difference 0.17 points, *P*=.032). Differences in ratings by sex or educational attainment were not statistically significant at either time point. Most participants 80+ years of age (28/37, 76%) agreed or strongly agreed with survey items at long-term follow-up, however their ratings were significantly lower than participants in younger age groups at both time points.

**Conclusions:**

Study results indicate it is feasible for older adults (70-90+ years of age) to use PAMs when self-tracking their physical activity, and provide a basis for developing recommendations to integrate PAMs into promotional efforts.

**Trial Registration:**

Clinicaltrials.gov NCT02433249; https://clinicaltrials.gov/ct2/show/NCT02433249 (Archived by WebCite at http://www.webcitation.org/6gED6eh0I)

## Introduction

Physical activity (PA) confers health benefits across older adult populations, including those with chronic conditions, and supports their autonomy and quality of life as they age [1]. Fewer than 1 in 5 older adults engage in recommended levels of physical activities [2]. Commercially available physical activity monitors (PAM) have the potential to facilitate PA and thus become a valuable adjunct to increasing PA levels [3]. To date, owners of such PAMs are predominantly young technology enthusiasts and those interested in confirming their fitness levels [4]. Design and marketing strategies for PAMs tend to track these patterns. Thus, it is not surprising that few older adults own [5] or use [6] such devices. However, the personalized data and support provided by PAMs could be beneficial to older adults.

### Background

Many older adults currently track their exercise, weight, or diet using pencil and paper or their memory [6]. Self-tracking PA (a form of self-monitoring outcome behavior) is a behavioral-change technique shown to increase older adults’ self-efficacy for exercise [7] and their initiation of new PA behavior [8]. Commercially available activity monitors have the potential to augment the PA self-tracking practices of older adults.

There is growing evidence refuting the perception that older adults are not interested in, or do not have the ability to use, technology in their everyday life. Older adults report having interests in using technology, particularly when it is easy to use, convenient (eg, fits well into daily routines), and benefits their health and wellness [9]. In a 2014 survey of older adults, 78% report using mobile phones (22% smartphones); 62% report using tablets; and 59% report using the Internet, primarily to communicate with family and friends, to shop, and to obtain health information [10].

Given that older adults practice self-tracking and have interests in (and the abilities to use) technology, researchers have begun to examine their acceptance of PAMs. Initial findings suggest that older adults evaluate PAMs positively in terms of comfort, ease of installation, and usefulness [11]. Participants also report having an interest in purchasing and using PAMs in the future [11,12]. Although promising, these initial studies assessed the experiences of older adults over short durations, ranging from three days to three weeks, which may not represent long-term experiences. For example, 6-12 months after purchasing PAMs, many younger owners report abandoning them [5]. Another limitation of initial studies is that participants were, on average, in their mid-60s. The experiences of 65-year-olds may not represent the experiences of people in their 70s, 80s, and 90s. Further evaluation of older adults’ long-term experiences with PAMs will build on this research and provide a basis for developing recommendations regarding the use of PAMs to facilitate PA tracking practices in this population.

### Study Objectives

The purpose of this study was to assess the short-term (10 week) and long-term (8 month) experiences of community-dwelling older adults using a popular, commercially available PAM (Fitbit One) to self-track their PA. The technology acceptance model (TAM) guided this assessment. The TAM posits that a person’s intention (*acceptance*) to use a new technology such as a PAM depends on their perceptions of its *ease-of-use* and its *usefulness* [13]. Research questions at the short-term and long-term assessment time points were:

1. Do older adults believe that PAMs are easy to use?

2. Do older adults believe data provided by a PAM is valuable and useful for self-monitoring and supporting their PA?

3. Do older adults with experience using a PAM intend to continue using it to track their PA?

In addition to these TAM-guided research questions, we sought to determine the extent to which participants’ perceptions and evaluations of PAMs differ between men and women, by level of educational attainment, and by age.

## Methods

### Design

This prospective study included participants from a randomized controlled trial (RCT) testing a fall-reducing PA promotion intervention in community-dwelling older adults [14]. All study participants received a PAM (Fitbit One) to facilitate their self-tracking practices throughout the 8-month study. Participants’ experiences were assessed after two main phases of the RCT: an intervention phase and a follow-up phase. During the 10-week intervention phase, participants had regular contact with intervention staff and structured support for using their PAMs. During the six-month follow-up phase, participants were left to use their PAMs independently. Thus, the respective 10-week and 8-month assessments represent short-term experiences using a PAM with structured support and longer-term experiences using a PAM without structured support. The Institutional Review Board at the University of Minnesota (UMN) approved the study protocol.

### Participants

The 95 participants in this study were community-dwelling older adults recruited via newspaper advertisements and flyers, which were placed in locations frequented by older adults living in Minneapolis, Minnesota between April and August 2014. Eligibility criteria included being 70 years old or greater, having the ability to walk, having the ability to speak English, having levels of PA below recommended guidelines [1], and not having a diagnosis of dementia. As the parent RCT was a pilot study, a convenience sample was used. Participants were given an incentive of $20 to complete each interview (3 for the current study) and invited to keep their PAM after completing the study.

### Procedures

Fitbit Ones were used in this study for four reasons. First, their displays provide data about several PA indicators (eg, steps, floors, distance, activity bouts). This level of detail makes them usable as stand-alone PAMs for individuals who cannot, or prefer not to, access the Internet. Second, there is an emerging body of evidence regarding the accuracy of the Fitbit One to sense PA [15-17] and estimate energy expenditure [11,18] in older adults when compared to observations of step counts and other accelerometers used in research. Third, research platforms are available that securely aggregate, store, analyze, and export de-identified data from many PAM wearers [19]. Finally, features of the Fitbit One used in this study were consistent with the theoretical basis [20] and intervention strategies used in the parent RCT (eg, selectively share data, individualize PA goals, self-monitor behavioral outcomes).

Participant feedback during the first few weeks of the study informed the refinement of PAM-related protocols. The first refinement was to decrease the manufacturer-set goals within the PAM (eg, 10,000 steps, 5 miles) to be safe and more relevant for older adult populations (eg, 1500 steps, 0.5 miles). The second was to use display characteristics most valued by older adults in this study, including greetings (eg, “Hi Tom.”), chatter (eg, “You rock.”, “Ready?”), steps, distance, and a flower that grows with continuous activity. Finally, participants were given the option to use graphing worksheets developed for those wanting to visualize their PA trends, but without access to (or willingness to use) the Internet for visualizing their PA dashboards.

Participants received a basic orientation when first enrolling into the RCT, assistance with troubleshooting for the next 10 weeks, and limited assistance after that. Upon enrollment, participants received a 15-30 minute basic orientation introducing the purpose of the PAM and demonstrating its core functions: charging, wearing, and reading displays. Participants were encouraged to demonstrate the skills they just learned and to ask questions. Individuals were also encouraged to use the project telephone number when in need of troubleshooting assistance or advanced orientation for operations, such as changing personal goals within the PAM and using a smartphone app to visualize their data. After being in the RCT for 10 weeks, participants received only limited troubleshooting assistance via the project telephone number. Approximately 10 individuals called during this time-frame with questions and challenges solvable by phone. Examples include getting their PAM out of its sleep mode, replacing lost or damaged PAMs, and synchronizing difficulties.

Research assistants (RA) collected data during one-on-one interviews using standardized procedures at three time points. The first time point was within one week of enrolling into the RCT; RAs administered a baseline characteristic questionnaire comprised of clinical and demographic variables. The second and third time points were 10 weeks and 8 months post-RCT enrollment; RAs administered technology surveys. Research Electronic Data Capture (REDCap) is a secure, web-based application designed to support data capture and management for research studies hosted at the UMN, that was used to collect and manage all data [21].

### Measures

Baseline characteristic questionnaires included the following demographic and clinical variables: self-reported age, sex, educational attainment, race, ethnicity, technology experience, and health conditions. The 10-item technology survey, adapted from previous usability and acceptability studies [13,22] addressed the 3 TAM domains (perceived *ease-of-use*, perceived *usefulness*, and *acceptance*), using a 5-point Likert scale ranging from 1 (strongly disagree) to 5 (strongly agree). Perceived *ease-of-use* is the degree to which a person believes technology use is free of effort. This TAM domain, linked to the research question asking if older adults believe that PAM are easy to use [13,22], was measured using five items (Cronbach alpha=.80, see items 1-5 in Multimedia Appendix 1). Perceived *usefulness* is the degree to which a person believes a technology provides useful information. This TAM domain, linked to the second research question about older adults' beliefs regarding the value and usefulness of data provided by a PAM [13], was measured using four items (Cronbach alpha=.80, see items 6-9 in Multimedia Appendix 1). *Acceptance* is the degree to which a person intends to use technology. This TAM domain, linked to the third research question regarding whether older adults intend to continue using a PAM to self-track their PA [12], was measured using one item (see item 10, Multimedia Appendix 1).

### Data Management and Analysis Plan

The online designer within REDCap enabled the creation of user-friendly case report forms for the baseline characteristic questionnaire and the technology survey (see Multimedia Appendix 1) with real-time data entry validation (completion), audit trails, and the ability to schedule participant interviews 10 weeks and 8 months after study enrollment. Research assistants entered real-time data into a REDCap case report forms using tablet computers during interviews with participants.

De-identified data from aggregated case report forms were exported into SPSS 22 and analyzed in two phases. First, univariate analyses were conducted to summarize participants’ baseline characteristics and survey scores. The distributions of individual mean scores within each survey domain, assessed by visual inspection of histograms, were not normally distributed. Therefore, the second phase of analysis used non-parametric inferential statistics. Median differences in ratings between 10-week and 8-month time points were analyzed using the Wilcoxon signed-rank test, a non-parametric analog to the paired samples t-test. The Mann-Whitney U and the Kruskal-Wallis tests, non-parametric analogs to independent-samples t-tests and one-way analysis of variance, were used to test median differences between multiple groups (eg, sex, age, education) at the same time point. Groupings for educational attainment (high school, at least some college, or college graduate) and age (70-74, 75-79, or 80+) are consistent with Smith’s survey of older adults and technology use [10]. Bonferroni corrections were made when multiple hypotheses were tested simultaneously. Adjusted P-values are presented.

## Results

### Baseline Demographic Characteristics

Ninety-five community-dwelling older adults participated in the study, most of whom were women (71/95, 75%) with some college education (68/95, 72%), ranging in age from 70 to 96 years of age (mean 79.8, SD 6.8). See Table 1 for baseline characteristics.

**Table 1:** Baseline characteristics of study participants (N=95).

		n (%)
Age	70-74	31 (33%)
	75-79	25 (26%)
	80+	39 (41%)
Race	African American	23 (24%)
	White	72 (76%)
Sex	Female	71 (75%)
	Male	24 (25%)
Education	Less than High School	3 (3%)
	High School	21 (22%)
	Some College	24 (26%)
	College Graduate	44 (46%)
	Refused	3 (3%)
Prior Technology Experience	Used Smart Phone	9 (9%)
	Used Laptop or tablet	31 (33%)
	Used PAM	1 (1%)
Chronic Conditions	Diabetes	27 (28%)
	Heart Disease	32 (34%)
	Lung Disease	9 (10%)
	Arthritis	65 (68%)

### Ease-of-use

**Figure 1:**
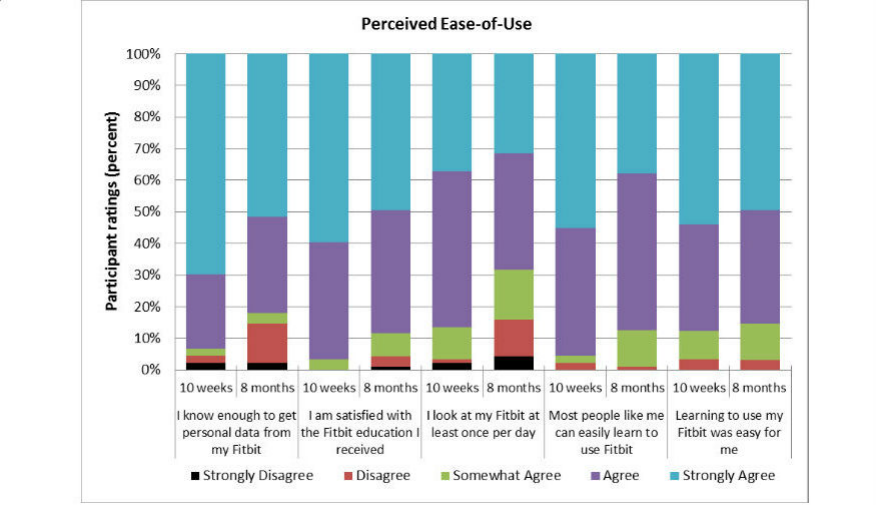
Perceived Ease-of-Use of Technology at 10 weeks and 8 months.

Overall, participants rated the *ease-of-use* domain positively at 10 weeks (median 4.60/5) and 8 months (median 4.20/5). Median differences between these time points were statistically significant as indicated by a Wilcoxon signed-rank test, *P*<.001. Follow-up tests revealed ratings of three items dropped significantly: *Most people, like me, could easily learn to use a Fitbit One* (median difference 0.23 points, *P*=.003); *I look at my Fitbit One at least once per day (*median difference 0.23 points, *P*=.001); and *I have sufficient information to help me get my personal data from my Fitbit One* (median difference 0.21 points, *P*=.008). Most participants (78/95, 82%) continued to agree or strongly agree with items in this domain after 8 months of ownership. Figure 1 illustrates the distribution of participant ratings (range 1-5) for the five items within this domain at 10 weeks and 8 months.

### Usefulness

**Figure 2:**
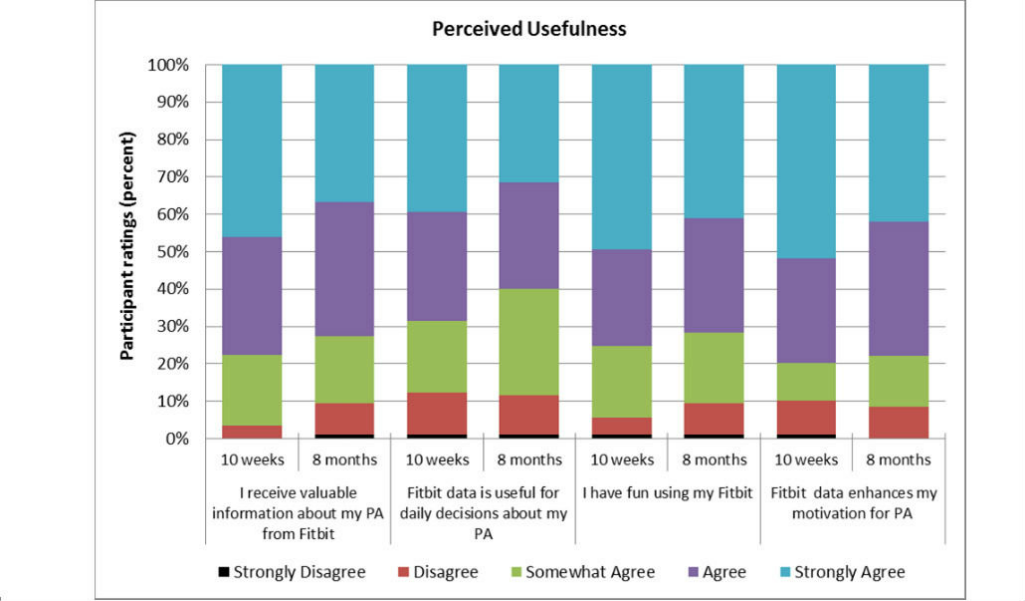
Perceived Usefulness of Technology at 10 weeks and 8 months.

Overall, participants rated the perceived *usefulness* domain positively at 10 weeks (median 4.13/5) and 8 months (median 3.98/5). Median differences between these time points were not statistically significant as indicated by a Wilcoxon signed-rank test (*P*=.19). Most participants (65/95, 68%) continued to agree or strongly agree with items in this domain after 8 months of ownership. Figure 2 illustrates the distribution of participant ratings (range 1-5) for the four items within this domain at 10 weeks and 8 months.

### Acceptance

**Figure 3:**
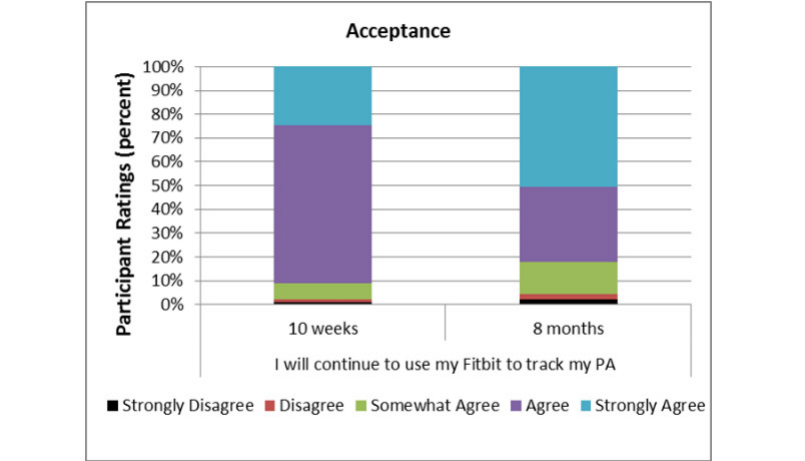
Technology Acceptance at 10 weeks and 8 months.

Overall, participants rated the *acceptance* domain positively at 10 weeks (median 4.54/5) and 8 months (median 4.25/5). Median differences in ratings between these time points were statistically significant as indicated by a Wilcoxon signed-rank test, *P*=.025. Although ratings declined, most participants (82/95, 86%) continued to agree or strongly agree with this item after 8 months of use. Figure 3 illustrates the distribution of participant ratings (range 1-5) of this one-item domain.

### Participant Ratings by Sex, Education Attainment, and Age

Differences between men and women’s median ratings of the three survey domains were not statistically significant as evidenced by Mann-Whitney U tests for *ease-of-use* at 10 weeks (median difference 0.11, *P*=.91) and 8 months (median difference 0.40, *P*=.65); *usefulness* at 10 weeks (median difference 2.00, *P*=.14) and 8 months (median difference 1.02, *P*=.19); and *acceptance* at 10 weeks (median difference 0.40, *P*=.77) and 8 months (median difference 0.23, *P*=.27).

Differences in ratings between participants in the three education attainment groups (high school, some college, or college graduates) were not statistically significant, as evidenced by the Kruskal-Wallis test of mean rank differences for: *ease-of-use* at 10 weeks (range of mean ranks 38-49, *P*=.21) and 8 months (range of mean ranks 44-49, *P*=.55); *usefulness* at 10 weeks (range of mean ranks 40-47, *P*=.51) and 8 months (range of mean ranks 41-48, *P*=.58); and *acceptance* 10 weeks (range of mean ranks 43-47, *P*=.56) and 8 months (range of mean ranks 40-49, *P*=.33).

Differences in ratings between participants in the three age groups were statistically significant at 10 weeks for all three survey domains: *ease-of-use*, χ^2^_2_ = 13.83, *P*=.002; *usefulness*, χ^2^_2_ = 20.41, *P*<.001; and *acceptance* χ^2^_2_ = 12.52, *P*=.002. Follow-up pairwise comparisons revealed lower ratings for the *ease-of-use* domain among participants in the 80+ age group (mean rank 33) compared to those in the 70-74 age group (mean rank 53, *P*=.005), and those in the 75-79 age group (mean rank 55, *P*=.006). Participants in the 80+ age group also rated the *usefulness* domain lower (mean rank 31) than those in the 70-74 age group (mean rank 51, *P*=.004), and the 75-79 age group (mean rank 62, *P*<.001). Finally, participants in the 80+ age group also rated the *acceptance* domain lower (mean rank 36) than those in the 70-74 age group (mean rank 54, *P*=.005) and the 75-79 age group (mean rank 50, *P*=.019).

Differences in ratings between participants in the three age groups at 8 months were also statistically significant for *ease-of-use*, χ^2^_2_ = 6.89, *P*=.032; *usefulness*, χ^2^_2_ = 6.90, *P*=.032; and *acceptance* χ^2^_2_ = 13.56, *P*=.001. Follow-up pairwise comparisons revealed lower ratings for the *ease-of-use* domain among participants in the 80+ age group (mean rank 39) compared to those in the 70-74 age group (mean rank 55, *P*=.036). Analyses also revealed lower ratings for *acceptance* among participants in the 80+ age group (mean rank 37), compared to those in the 70-74 age group (mean rank 59, *P*=.001). However, differences between age groups in ratings of the *usefulness* domain were not statistically significant (*P*>.067). Although ratings among participants in the 80+ age group were lower compared to younger age groups, most participants in this oldest age group (28/37, 76%) agreed or strongly agreed that the PAM was *easy to use*, *useful*, and *acceptable* after 8 months of ownership.

## Discussion

The purpose of this study was to assess older adults’ short and long-term experiences using PAM to self-track their PA. Overall, older adults evaluated the PAM as *easy to use*, *useful*, and *acceptable* with and without structured support. Although the *ease-of-use* and *acceptance* ratings decreased over time, and ratings were lower among those who were >80 years old compared to younger age groups, most ratings were positive.

Short-term (10 week) participant ratings across all three survey domains (perceived *ease-of-use*, *usefulness*, and *acceptance*) were positive and consistent with previous research [11,12]. Longer-term (8 month) follow-up ratings were mostly positive, but there were no comparable studies. Researchers at Endeavor Partners surveyed PAM owners (mostly younger adults) and found that more than 30% have abandoned their device within 8 months [5]. To compare, we estimated the 8-month abandon rate in this study by calculating the percentage of participants who either disagreed or strongly disagreed with the statement, “I will continue to use my Fitbit One to track my PA*”*, which was 4%. Lower rates of abandonment by participants in this study may have been, in part, because of the structured support they received during the first 10 weeks of the study. It is also possible that older adults, more than younger populations, find PAMs have long-term utility.

Lower survey ratings by participants >80 years old, observed at both time points, are consistent with this age group’s response to other technology-adoption surveys (all aspects of digital life) [10]. One possible explanation may be that there are age-related differences in individual technology adoption rates or initial decisions about using technology. Participants aged 70-79, more than participants >80 years, may have been *early adopters* or had lower levels of uncertainty when first introduced to Fitbit Ones [23]. These differences might also reflect a need for more time among participants >80 years old to learn new technology, compared to younger participants. Older adults have reported that they need someone to walk them through the process of using a new technology [10], and that social learning (learning new technology with at least one other person) is easier than learning alone with the aid of a manual [24]. These findings validate the need for limited support, especially while individuals are first learning PAM characteristics and making decisions about how the technology might be relevant to personal health-related values.

### Limitations

This study had several limitations. The experiences of older adults in this sample were assessed using just one popular PAM; ratings of PAM usage and acceptance may vary over time and by model. A second limitation was that PAM usage in this study was in a context of high support, at least during the initial 10-week period. This level of support exceeds what new owners of commercially available PAMs typically acquire, such as tailoring manufacturer-set goals within PAMs to be consistent with typical goals of older adults, and providing participants who preferred not to use the Internet with tools to aid their visualizations of aggregate data. A third limitation is that TAM, the conceptual model used in this study, posits that the two main drivers of technology acceptance are perceived *usefulness* and perceived *ease-of-use*. Additional drivers may be important to consider in older adult populations, such as health benefits and emotional satisfaction [25,26]. Finally, the sample in this study was not representative of the general US population of older adults. The proportion of female participants in this study (71/95, 75%) was higher than that of older adults in the US population (59%), and the proportion of participants with college degrees (44/95, 46%) was higher than that of the US population (26%) [27].

### Future Research

The findings of this study support future research to examine the facilitating potential of PAM activity trackers on older adults’ PA, and hint at opportunities for new designs to optimize meaningful use. It is unlikely that ownership of a PAM, alone, will elicit changes in PA behavior [3]. Thus, examining the facilitative potential of PAMs will be most informative when using them as a medium for testing various engagement strategies [5] and behavioral change techniques [28]. Just as ownership will not drive behavior, nor will the designs of these PAMs. However, some tailoring of characteristics such data cuts and data visualization might improve the experiences of older adults using PAMs [29]. For example, visualization possibilities for older adults might be simplified, particularly within a single device. It will be worthwhile to explore how display options on PAMs, such as Fitbit One, might display relevant data cuts of weekly PA trends (eg, small bar graphs or other visualizations). Another possibility is to explore ways in which older adults can easily and selectively share their personal PA data (not including social media venues) with important others such as family, friends and healthcare providers. Exploration of new PAM designs tailored for older adults will yield the most information when they include an understanding of their unique preferences, capabilities, and limitations, as well as sensitivity to what might undermine their individual potential. Such an understanding will require person-centered design strategies, as recommended by experts in the fields of human-computer interaction, psychology, and gerontology [25,26,30,31].

### Conclusion

This study provides valuable insights into older adults’ short and long-term experiences with a commercially available PAM for self-tracking PA, the Fitbit One. Older adults with little technology experience, low levels of PA, as well as diverse PA goals and abilities, found the PAM *easy to use* and *useful* for self-tracking their PA. Thus, despite design and marketing strategies of PAMs that primarily target younger populations, it is feasible for older populations (70-90+ years of age) to use PAMs in ways that support their unique PA goals. These results support the potential of PAM technology to facilitate PA in older adults and provide a basis for developing recommendations for promoting such use.

## Appendix

Multimedia Appendix 1 Technology Survey.

## Abbreviations

PA: physical activity
PAM: physical activity monitor
RA: research assistant
RCT: randomized control trial
REDCap: Research Electronic Data Capture
TAM: technology acceptance model
UMN: University of Minnesota
