# Supplementary material for: Older Adults’ Experiences Using a Commercially Available Monitor to Self-Track Their Physical Activity
Source: JMIR Mhealth Uhealth. 2016 Apr 13;4(2):e35. doi: 10.2196/mhealth.5120 (PMC4848389; doi:10.2196/mhealth.5120)
Supplement: Multimedia Appendix 1 [file mhealth_v4i2e35_app1.pdf]

Confidential

Technology Usability and Acceptability

Record ID ______________________________

Technology usability and acceptability

The next ten questions ask about your opinion(s) of the personal activity monitors used in this study, Fitbits. We do not have interest or ownership in the Fitbit company. Your experiences using the Fitbit will help us in future research and practice.

	strongly disagree	disagree	somewhat agree	agree	strongly agree
I will continue to use my Fitbit™ to track (monitor) my physical activity	○	○	○	○	○
Learning to use my Fitbit™ was easy	○	○	○	○	○
Most people, like me, could easily learn to use a Fitbit™ to track (monitor) their physical activity	○	○	○	○	○
I receive valuable information about my levels of physical activity by using my Fitbit™	○	○	○	○	○
Information from my Fitbit™ is useful as I make daily decisions about my physical activities	○	○	○	○	○
Information from my Fitbit™ enhances my motivation for physical activity (e.g., walking, strength/ balance exercises)	○	○	○	○	○
I have fun using my Fitbit™	○	○	○	○	○
I look at my Fitbit™ at least once per day	○	○	○	○	○
I have sufficient information to help me get my personal data from my Fitbit™	○	○	○	○	○
I am satisfied with the education that I received for using my Fitbit™	○	○	○	○	○
